# Supplementary material for: Analysis of entropy on the European markets of energy and energy commodities prices
Source: PLoS One. 2025 Jan 16;20(1):e0315348. doi: 10.1371/journal.pone.0315348 (PMC11737706; doi:10.1371/journal.pone.0315348)
Supplement: Attachment — Submitted filename: Response to Reviewers .docx [file pone.0315348.s004.docx]

Dr Daniel Papla

November 11, 2024

Subject: manuscript ID: PONE-D-24-36364

Dear Sirs,

Thank you for allowing us to submit a revised draft of our manuscript. We appreciate the time and effort that you and the reviewers have dedicated to providing valuable feedback on our manuscript. We have incorporated changes to reflect most of the suggestions provided by the reviewers.

**Reviewer 1**

Thank you for your pertinent comments; the purpose and hypotheses were unclear. Following your suggestions, we have improved and clarified our purpose and hypotheses so that they are clear, and we have specified our scope of research.

**Reviewer 2**

Thank you for your insightful comments and the amount of work you put into making the language corrections, which we have considered. In addition, in accordance with the comments:

- we have corrected the research objective and hypothesis,

- we have explained the choice of method (Shanon entropy and why we do not use, for example, the Tsallis and Renyi methods – which are used in chemical and physical sciences, and Shanon derives from information theory) and the use of the Jems-Stein estimator and why they seem to be appropriate in this case

- we have added and expanded the definitions of terms and the methodology and/or corrected the colloquial or illegible formulations.

- we have expanded the robustness check

- we have corrected the conclusions and added future studies.

- Comparison of results using other methods, such as the Hurst coefficient, econometric methods (ARIMA, GARCH, etc.) and different methods of examining entropy is in the course of our research. We hope it will be presented in subsequent publications.

Sincerely,

Daniel Papla, PhD
